# Characterization of 3D-printed PLA parts with different raster orientations and printing speeds

**DOI:** 10.1038/s41598-022-05005-4

**Published:** 2022-01-19

**Authors:** Mohammad Reza Khosravani, Filippo Berto, Majid R. Ayatollahi, Tamara Reinicke

**Affiliations:** 1 Chair of Product Development, University of Siegen, Paul-Bonatz-Str. 9-11, 57068 Siegen, Germany; 2 Department of Mechanical and Industrial Engineering, Norwegian University of Science and Technology (NTNU), 7491 Trondheim, Norway; 3 Fatigue and Fracture Research Laboratory, Center of Excellence in Experimental Solid Mechanics and Dynamics, Iran University of Science and Technology, Narmak, 16846 Tehran, Iran

**Keywords:** Engineering, Mechanical engineering, Mechanical properties

## Abstract

Fabrication based on additive manufacturing (AM) process from a three-dimensional (3D) model has received significant attention in the past few years. Although 3D printing was introduced for production of prototypes, it has been currently used for fabrication of end-use products. Therefore, the mechanical behavior and strength of additively manufactured parts has become of significant importance. 3D printing has been affected by different parameters during preparation, printing, and post-printing processes, which have influence on quality and behavior of the additively manufactured components. This paper discusses the effects of two printing parameters on the mechanical behavior of additively manufactured components. In detail, polylactic acid material was used to print test coupons based on fused deposition modeling process. The specimens with five different raster orientations were printed with different printing speeds. Later, a series of tensile tests was performed under static loading conditions. Based on the results, strength and stiffness of the examined specimens have been determined. Moreover, dependency of the strength and elastic modulus of 3D-printed parts on the raster orientation has been documented. In the current study, fractured specimens were visually investigated by a free-angle observation system. The experimental findings can be used for the development of computational models and next design of structural components.

## Introduction

Additive manufacturing (AM) also known as three-dimensional (3D) printing covers a set of techniques which utilized layer-by-layer concept to fabricate components. 3D printing technology is vastly used in different applications, such as aerospace, automotive, electronics, construction, and medicine, and healthcare monitoring^1–6^. Due to favorable properties of 3D printing technology, it has attracted great interest in various technological sectors.

Based on the documented applications 3D printing, reduction in time and cost by eliminating expensive manufacturing equipment, and possibilities on easy fabrication of geometrically complex components have been considered as important advantages of 3D printing technology. Moreover, in this rapid prototyping process, large reduction of waste material can be achieved, because manufacturing tools are not required, and filed prints can be recycled in an easy and fast process. Production of light structural components with desired weight and utilizing multiple materials at the same time are advantages of 3D printing compared to traditional manufacturing processes.

As applications of AM have been significantly increased, different engineering aspects have been studied in this field^7–10^. Although 3D printing was introduced for production of prototypes, it is being used to fabricate final products. In this case, 3D-printed parts and structural elements might be subjected to special loading or environmental conditions. Therefore, it is necessary to investigate mechanical behavior of these components. In this context, several studies have been conducted to determine response of 3D-printed materials to different loading conditions, such as bending, tensile, and torsion^11–13^. For instance, in Ref.^14^ dynamic behavior of 3D-printed reinforced composites was investigated. In detail, fused filament fabrication process was used to produce short carbon-fiber-reinforced composite specimens which are subjected to dynamic loading by a Hopkinson bar. The obtained results indicated that infill density has important role in deformation and strength, but low density and high density infills are cost effective compared to solid components.

Considering applications of 3D-printed components, their structural integrity become an important issue. Hence, fracture behavior of these parts are investigated in several studies^15–20^. In Ref.^21^ fracture behaviors of 3D printed components have been reviewed. In previous studies, fracture mechanics approach was used for characterization of additively manufactured components^22–26^. For instance, in Ref.^27^ effects of filament-scale geometric features have been investigated. More in deep, 3D-printed compact tension test coupons were examined with different fiber directions. The obtained results confirmed that higher strength is achievable with loading in the filament direction. Moreover, fractography indicated that interfacial weakness leads to anisotropy and change mechanical performance of the component.

Parallel to the experimental tests, numerical models and different artificial intelligence approaches have been used to investigate performance characteristics of 3D-printed components. For instance, in Ref.^28^ back propagation neural network was used to predict printable bridge length in fused deposition modeling (FDM) process. Recently, in Ref.^29^ we have reviewed applications of machine learning in prediction of mechanical behavior of 3D-printed parts. In order to enhance strength and improve mechanical performance of 3D-printed components, different attempts have been made^30–33^. For example, in Ref.^31^ annealing was performed to increase strength of 3D-printed parts. In this context, polymeric materials with different carbon fiber reinforced polymers were utilized to print dog-bone specimens based on material extrusion technique. According to the experimental practices, it has been concluded that annealing is a suitable post-processing technique to enhance interlayer tensile strength of 3D-printed composites. Since, mechanical behavior of 3D-printed components depends on various parameters, a significant amount of attempts has been made in this field^34–37^. Recently, in Ref.^38^ physicochemical and mechanical behavior of PLA matrix in 3D printed composite was investigated. At the same time, in Ref.^39^ FDM process was used to prepare specimens and effects of printing parameters such as nozzle temperature and layer thickness on flexural properties and impact strength of specimens were investigated.

In the current study, we investigated effects of two printing parameters on strength of 3D-printed parts: (a) raster orientation and (b) printing speed. To this aim, test coupons with various raster directions were printed with different speeds. We used PLA bioepolymer with grade of 4043D. Currently, PLA with different grades are used in 3D printing, and results of tests on a particular grade cannot be used for analysis of other grades, therefore, separate research studies are required to determine exact behavior of each grade. Here, a series of tensile tests were conducted and mechanical behavior and strength of the examined parts were documented. Although influence of printing parameters on the mechanical behavior of 3D-printed components have been discussed in some previous studies, to the best of the authors knowledge, experimental tests and two different theoretical formulas are not used on a particular research work with this grade of material which is investigated in the present study. Since creating a strong and dimensionally stable parts have been a challenging goal of FDM technology, study effect of manufacturing parameters on 3D-printed parts is a necessity.

## Results and discussion

**Figure 1:**
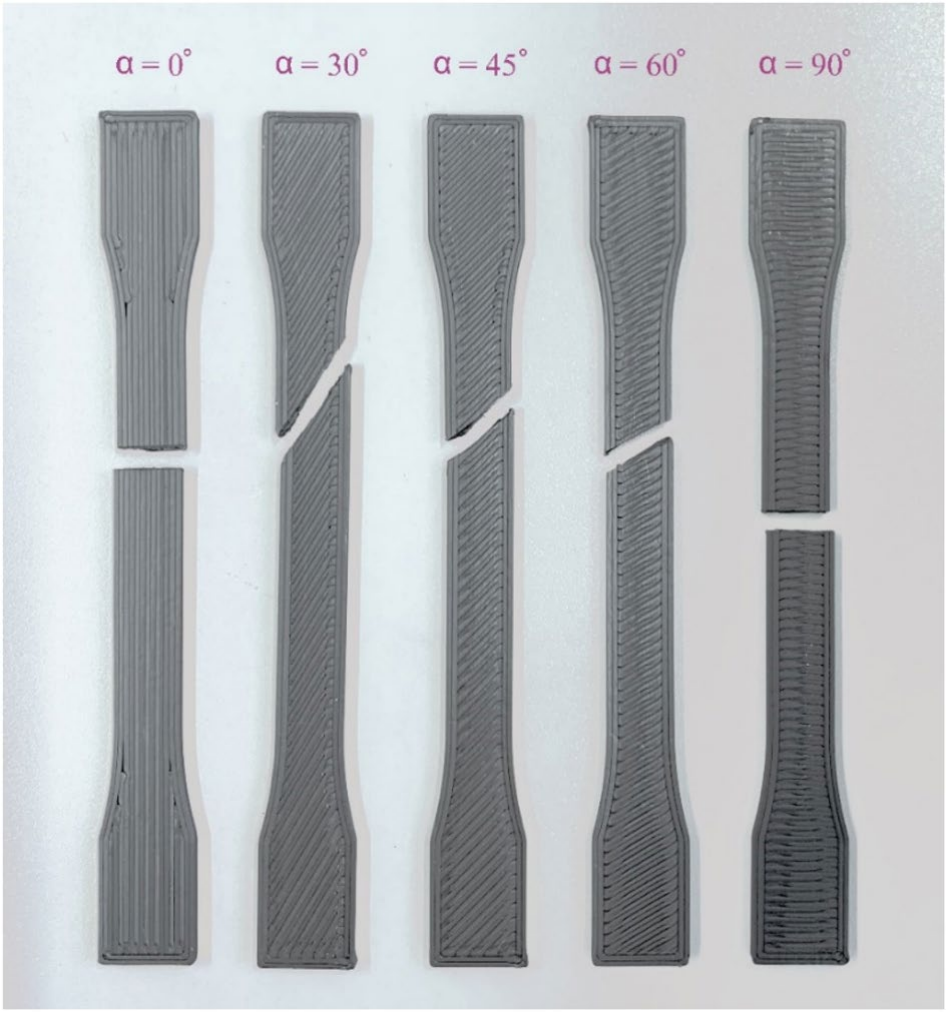
Fractured specimens with different raster directions, printed at 20 mm/s.

**Figure 2:**
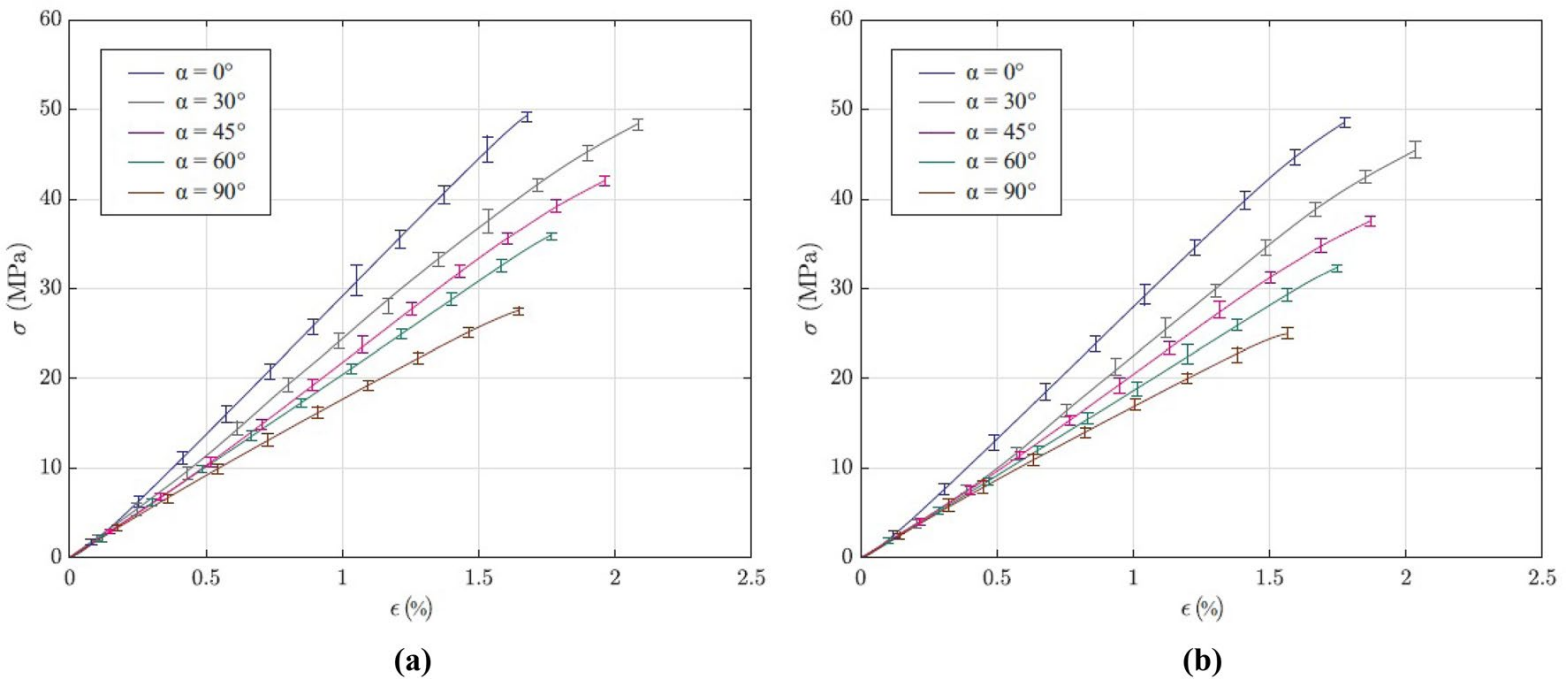
Stress–strain curves of the tested specimens, printed with different speeds: (**a**) 20 mm/s; (**b**) 80 mm/s.

Experimental practices confirmed that there was a sudden failure in the specimens after reaching the maximum stress. We documented rather brittle behavior in the examined specimens. In Fig. 1 a close-up pictures of the fractured specimens illustrated. Based on the performed tests, the mechanical behavior of the test coupons are determined. In Fig. 2 stress–strain curves of the examined specimens are illustrated. According to the results, strength and stiffness depend on the raster direction and printing speed.

In the present study, each sample set consisted of six specimens for a given group of process parameters (raster direction and printing speed), with a total of 60 specimens. All specimens were printed using Ender-3 Pro 3D Printer. Figure 2a shows resulting stress–strain curves of the specimens with different raster directions which are printed at 20 mm/s. It can be seen that 0° and 90° specimens indicated highest and lowest strengths, respectively. Additionally, Fig. 2b shows stress–strain curves of the specimens printed at 80 mm/s. As can be seen, an increase in the raster direction has led to decrease in strength of the 3D-printed parts. It is noteworthy that printing speed has effect on the elongation of examined specimens, but this influence is small compared to effect of raster direction on the mechanical behavior of 3D-printed components.

**Figure 3:**
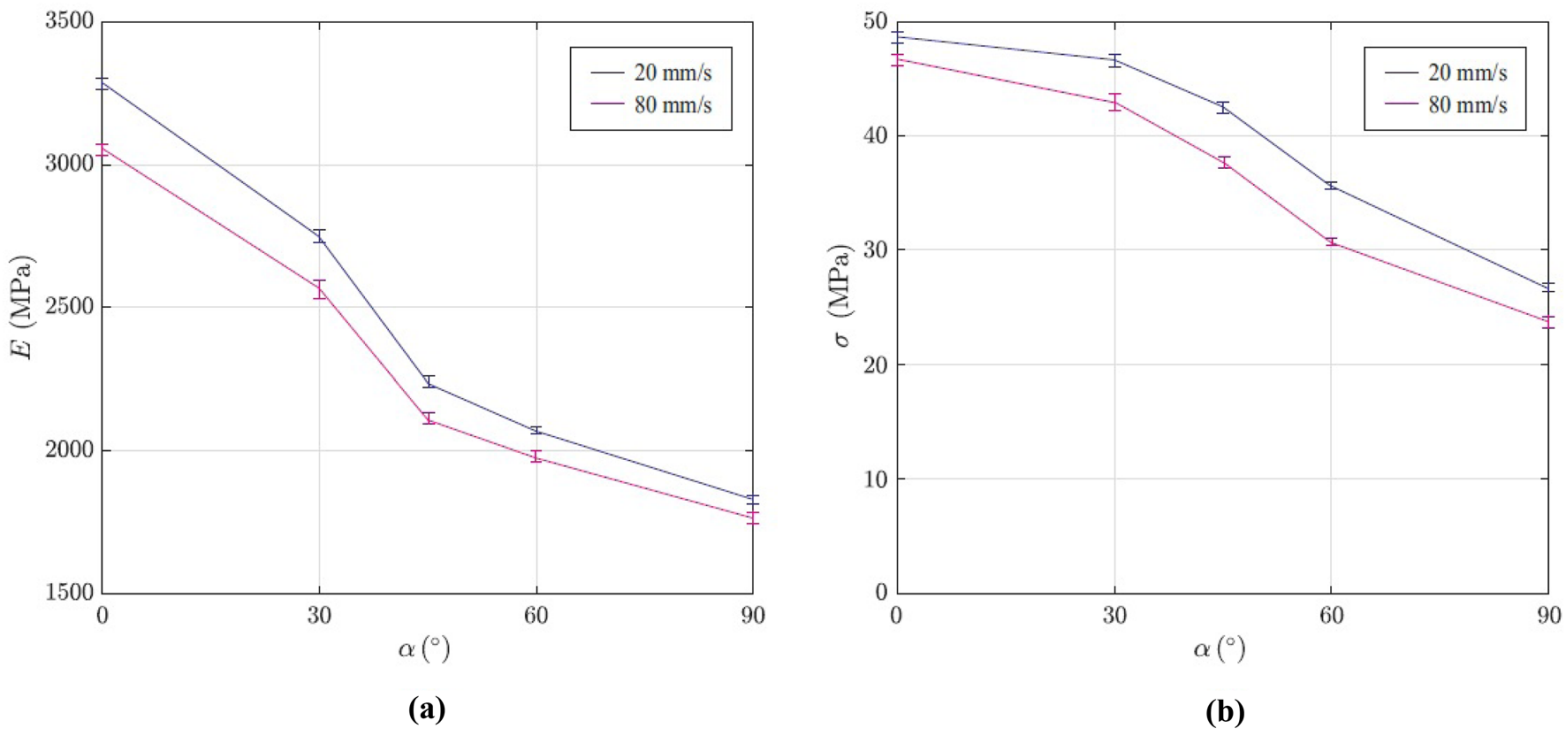
Dependency of (**a**) elastic modulus, and (**b**) tensile strength on the raster direction for the 3D-printed specimens.

According to the experimental findings, we have determined dependency of the elastic modulus and the tensile strength on the raster direction. In Fig. 3a relation between the raster direction and elastic modulus is depicted. As it is shown, an increase in the raster orientation has led to a decrease in elastic modulus of the examined 3D-printed specimens. Hence, 90° specimens presented lowest elastic modulus. Figure 3b shows dependency of tensile strength on raster orientation. The obtained results proved that tensile strength gradually decreased with an increase in the raster orientation.

Since FDM widely used in different applications, various aspects have been investigated in this process^40–42^. For instance, in Ref.^42^ finite element analysis of 3D printing based on FDM process was investigated. In detail, researchers studied impact of several parameters and modeling choices on the simulation findings. Here, based on the results, we concluded that raster direction has a significant effect on the strength of the specimens compared to printing speed. However, at the higher printing speed there is a shorter forming time which has a negative effect on the strength of printed specimens.

Analysis of fracture in the specimens indicated that the interlayer and in-layer fractures were occurred in the specimens after tensile tests. These fractures are defined as follows:

- Interlayer fracture: in this type of fracture, the angle $(\beta )$ between the materials layer and fracture surface of the materials is equal to zero. In this case, the materials layer would be intact after fracture.
- In-layer fracture: in this fracture, the angle $\beta$ is larger than zero and the materials layer would not be intact after the fracture.

**Figure 4:**
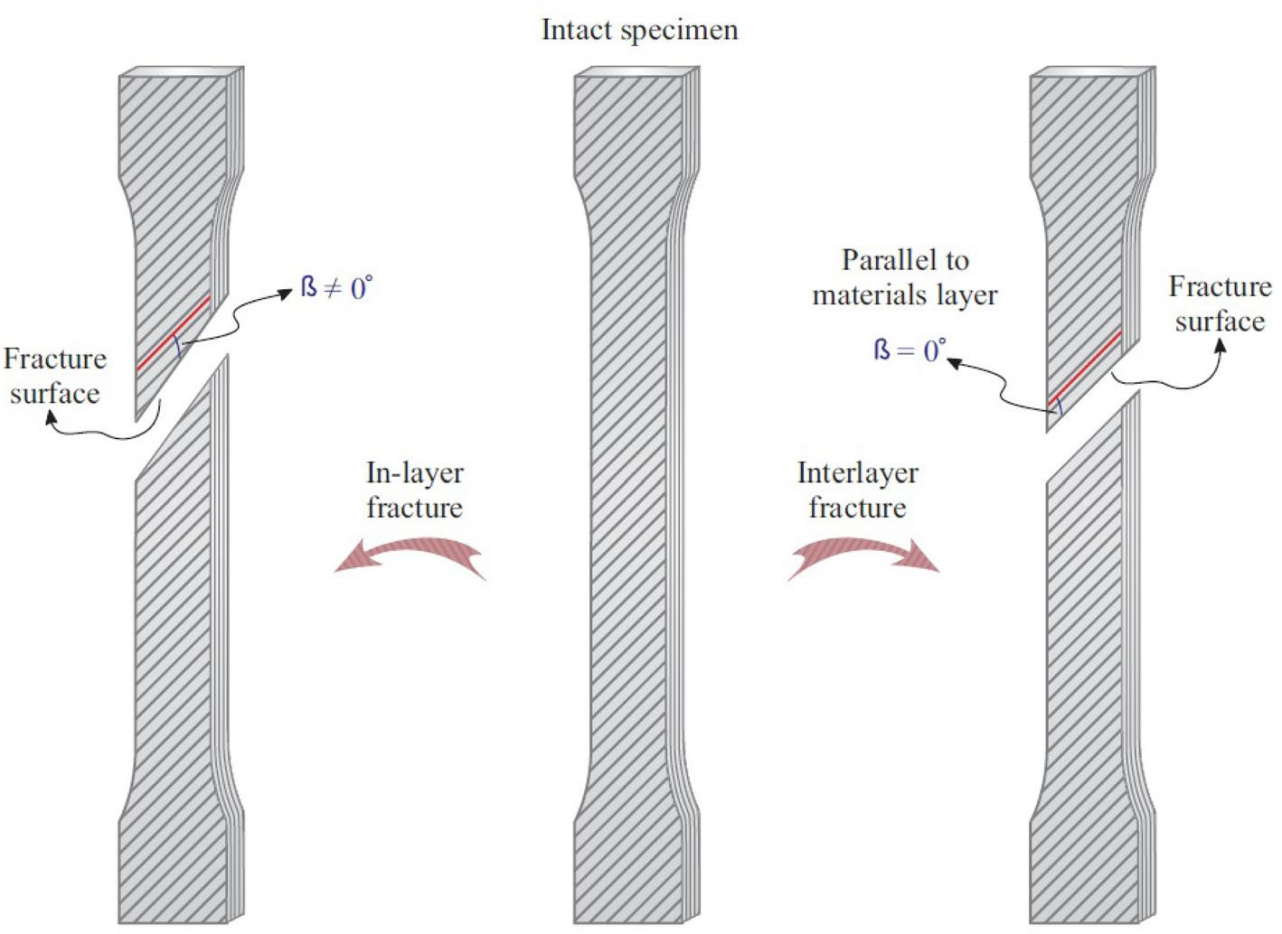
Schematics of interlayer and in-layer fractures.

In Fig. 4 interlayer and in-layer fractures are schematically shown. In the current study, both interlayer and in-layer fractures were occurred.

As mentioned earlier, the results showed that the UTS of 3D-printed parts decreased when raster direction increased from 0° to 90°. Here, we have considered the UTS of the 0° specimens as a standard (100%) and determine reduction coefficient of the examined parts with different raster directions as follows:

1 $$\text{Reduction \; coefficinet \; in \; UTS}=\frac{\text{UTS}}{\text{UTS \; in }\; {0}^{^\circ} \; \text{specimen}}.$$

Considering Eq. (1), reduction in the UTS of different specimens are summarized in Table 1. Experimental results indicated that the reduction in the UTS varies from 53.76 to 100%. In detail, specimen printed at 80 mm/s with 90° raster direction showed smallest reduction coefficient.

**Table 1:** Reduction in the UTS of different 3D-printed specimens.

Printing speed (mm/s)	Raster direction				
	0° (%)	30° (%)	45° (%)	60° (%)	90° (%)
20	100.00	95.83	87.51	72.91	58.33
80	100.00	92.47	81.72	68.81	53.76

**Figure 5:**
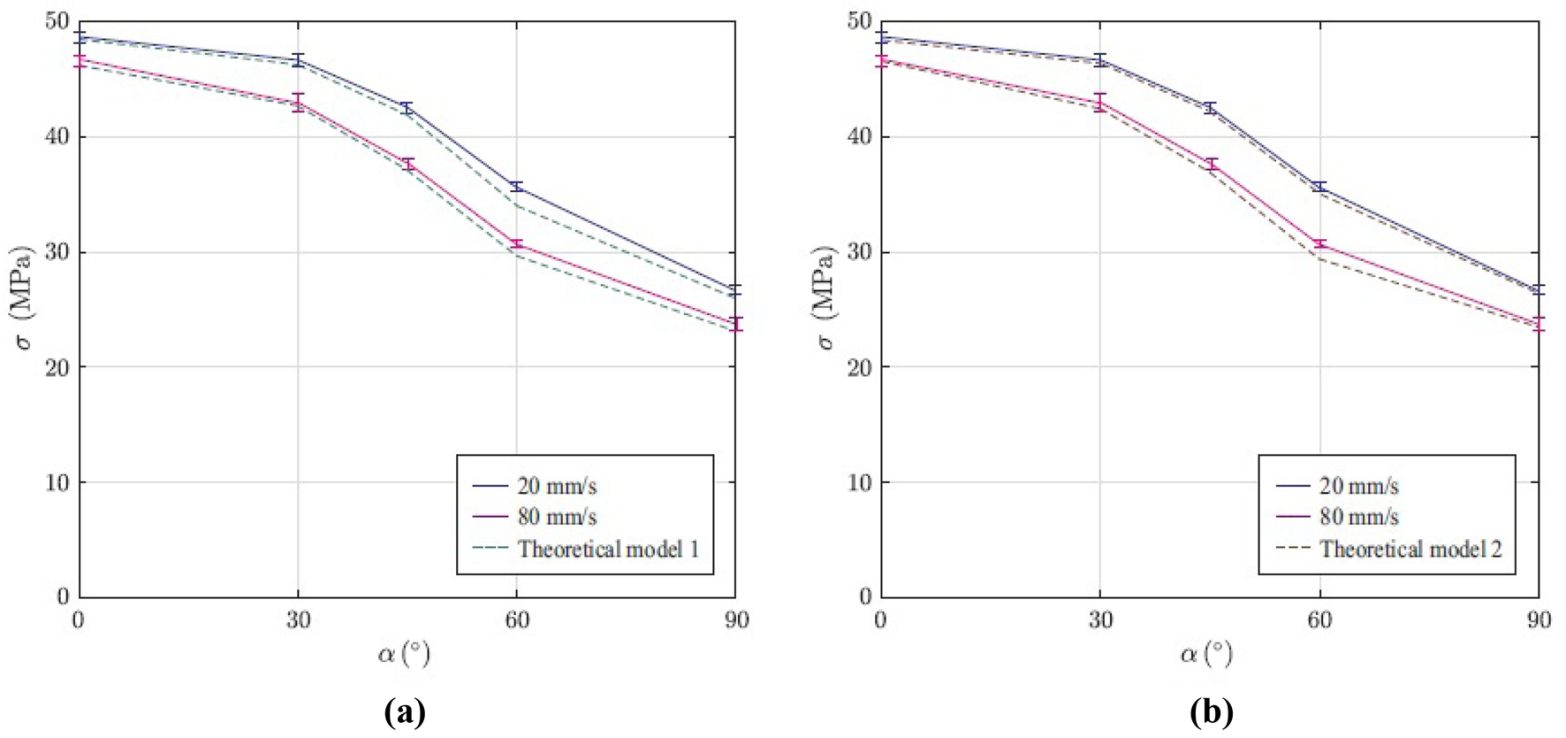
Comparison of the experimental results with (**a**) first and (**b**) second theoretical model.

Based on the current experimental results, theoretical models are evaluated. In Fig. 5 results of both theoretical models and experimental findings are compared. It can be clearly seen that the both theoretical models Eqs. (11) and (12) predicted the experimental results accurately.

Comparison of the experimental findings and theoretical models confirmed ability of both theoretical models in prediction of UTS of 3D-printed components.

**Figure 6:**
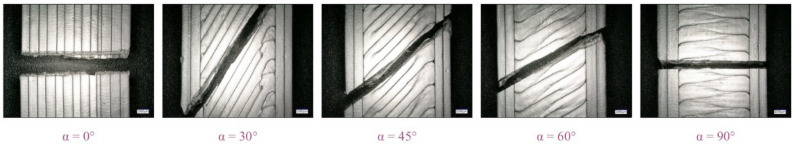
Fractured 3D-printed specimens with different raster orientations.

As there are different grades of PLA grades on the market, precise investigations are required to identify mechanical behavior of each grade. In the presented study, we used PLA bioepolymer with grade of 1043D. Based on the previous research by Bermundez et al.^43^ PLA with different grades showed different ultimate tensile strengths. Different types of manufacturing defects can significantly change the mechanical behavior of the components. As different defects and anomalies might be occurred during 3D printing process (e.g., overlap, missing extrudates, and offset), the specimens are visually investigated prior to the tensile test and we have not found any type of defect. After tensile tests, a series of fractographic examination was performed for analysis of the fracture. To this aim, a free-angle observation system was utilized for visual investigation of the fractured test coupons. Figure 6 shows fracture surfaces of examined specimens with different raster directions.

The visual inspection of fractured surfaces confirmed that in the 0° specimens the crack is oriented vertically to the raster direction. In 30°, 45°, and 60° specimens, the fractures are oriented with raster directions.

## Theoretical background

In the FDM process, 3D-printed parts are produced layer by layer, therefore, the parts can be considered as transverse isotropic materials. Indeed, each layer is isotropic materials in plane. Hence, the elastic constitutive relation of this material can be presented in the matrix form:

2 $$\left[\begin{array}{c}{\varepsilon }_{1}\\ {\varepsilon }_{2}\\ {\varepsilon }_{3}\\ {\varepsilon }_{4}\\ {\varepsilon }_{5}\\ {\varepsilon }_{6}\end{array}\right]=\left[\begin{array}{c}\begin{array}{ccc}{S}_{11} & {S}_{12} & {S}_{12}\\ {S}_{12} & {S}_{22} & {S}_{23}\\ {S}_{12} & {S}_{23} & {S}_{22}\end{array}\begin{array}{ccc} 0& 0& 0\\ 0& 0& 0\\ 0& 0& 0\end{array}\\ \begin{array}{ccc}0& 0& 0\\ 0& 0& 0\\ 0& 0& 0\end{array}\begin{array}{ccc} 2({S}_{22}-{S}_{23})& 0& 0\\ 0& {S}_{55}& 0\\ 0& 0& {S}_{55}\end{array}\end{array}\right] . \left[\begin{array}{c}{\sigma }_{1}\\ {\sigma }_{2}\\ {\sigma }_{3}\\ {\sigma }_{4}\\ {\sigma }_{5}\\ {\sigma }_{6}\end{array}\right].$$

As classically performed for the printed fiber^44^, we can assume the same mechanical behavior in two directions. In plane stress state $({\sigma }_{3}=0, {\tau }_{23}={\sigma }_{4}=0, {\tau }_{31}={\sigma }_{5}=0)$, the constitutive equation can be presented as follows:

3 $$\left[\begin{array}{c}{\varepsilon }_{1}\\ {\varepsilon }_{2}\\ {\gamma }_{12}\end{array}\right]= \left[\begin{array}{ccc}{S}_{11}& {S}_{12}& 0\\ {S}_{12}& {S}_{22}& 0\\ 0& 0& {S}_{55}\end{array}\right] \left[\begin{array}{c}{\sigma }_{1}\\ {\sigma }_{2}\\ {\tau }_{12}\end{array}\right].$$

In Eq. (3), the flexibility coefficient matrix $S$ has four independent elastic constants: ${S}_{11}, {S}_{2}, {S}_{22}$ and ${S}_{55}$. These constants can be presented as functions of elastic modulus, Poisson’s ratio, and shear modulus:

4 $${S}_{11}= \frac{1}{{E}_{1}} , {S}_{22}= \frac{1}{{E}_{2}} , { S}_{55}= \frac{1}{{G}_{12}} , {S}_{11}= \frac{{-\vartheta }_{21}}{{E}_{1}}= \frac{{-\vartheta }_{12}}{{E}_{2}}.$$

Equation (2) can also be presented as follows to obtain stiffness matrix Q:

5 $$\left[\begin{array}{c}{\sigma }_{1}\\ {\sigma }_{2}\\ {\tau }_{12}\end{array}\right]= \left[\begin{array}{ccc}{Q}_{11}& {Q}_{12}& 0\\ {Q}_{12}& {Q}_{22}& 0\\ 0& 0& {Q}_{55}\end{array}\right] \left[\begin{array}{c}{\varepsilon }_{1}\\ {\varepsilon }_{2}\\ {\gamma }_{12}\end{array}\right].$$

The constitutive equation in different directions of materials can be presented as follows:

6 $$\left[\begin{array}{c}{\sigma }_{x}\\ {\sigma }_{y}\\ {\tau }_{xy}\end{array}\right]= {T}^{-1} \left[\begin{array}{c}{\sigma }_{1}\\ {\sigma }_{2}\\ {\tau }_{12}\end{array}\right]= {T}^{-1}Q {\left[\begin{array}{c}{\varepsilon }_{1}\\ {\varepsilon }_{2}\\ {\gamma }_{12}\end{array}\right]=T}^{-1}Q{({T}^{-1})}^{T}\left[\begin{array}{c}{\varepsilon }_{x}\\ {\varepsilon }_{y}\\ {\gamma }_{xy}\end{array}\right],$$

where $T$ is the stress rotation matrix which can be expressed as follows with respect to $\theta$ which is the angle between the global axis and the material axis:

7 $$T=\left[\begin{array}{ccc}{cos}^{2}\theta & {sin}^{2}\theta & 2 sin\theta cos\theta \\ {sin}^{2}\theta & {cos}^{2}\theta & -2 sin\theta cos\theta \\ -sin\theta cos\theta & sin\theta cos\theta & {cos}^{2}\theta -{sin}^{2}\theta \end{array}\right].$$

Hill–Tsai anisotropic yield criterion can be written as:

8 $$\frac{{{\sigma }_{1}}^{2}}{{X}^{2}}+\frac{{{\sigma }_{2}}^{2}}{{Y}^{2}}-\frac{{\sigma }_{1}{\sigma }_{2}}{{X}^{2}}+\frac{{{\tau }_{12}}^{2}}{{W}^{2}}=1,$$

where $X,Y$, and $W$ are the ultimate tensile strength (UTS) in the principal axes of anisotropy. If there is only ${\sigma }_{x}$ in the loading direction of the test coupon, the stresses in the material reference system are as follows^45^:

9 $${\sigma }_{1}={\sigma }_{x}{cos}^{2}\theta , {\sigma }_{2}={\sigma }_{x}{sin}^{2}\theta , {\tau }_{12}={\sigma }_{x}sin\theta cos\theta .$$

Combination of Eqs. (8) and (9) leads to:

10 $${\sigma }_{x}={T}_{\theta }= {\left[\frac{{cos}^{4}\theta }{{X}^{2}}+\left(\frac{1}{{W}^{2}}-\frac{1}{{X}^{2}}\right){sin}^{2}\theta {cos}^{2}\theta +\frac{{sin}^{4}\theta }{{Y}^{2}}\right]}^{-1/2},$$

where $X$ and $Y$ denote the UTS of material in directions 1 and 2, respectively. Moreover, $W$ is ultimate shear strength of plane 12, and ${T}_{\theta }$ indicates the UTS of the load direction $({T}_{{0}^{^\circ }}=X$ and ${T}_{{90}^{^\circ }}=Y)$ From Eq. (9), in plane shear strength can be determined by:

11 $${W}_{HT}={\left[\frac{1}{{{T}_{\theta }}^{2}{sin}^{2}\theta {cos}^{2}\theta }-\frac{1}{{X}^{2}}.\frac{{cos}^{2}\theta }{{sin}^{2}\theta }-\frac{1}{{Y}^{2}}.\frac{{sin}^{2}\theta }{{cos}^{2}\theta }+\frac{1}{{X}^{2}}\right]}^{-1/2}.$$

Based on the two following theoretical models, the UTS of FDM 3D-printed parts can be determined with respect to the printing direction:

- First model: considering Eq. (10) and shear strength of composite $\left({W}_{sf}\right)$ the first model is as follows:
  12 $${T}_{\theta }={\left[\frac{{cos}^{4}\theta }{{X}^{2}}+\left(\frac{1}{{W}_{sf}}-\frac{1}{{X}^{2}}\right){sin}^{2}\theta {cos}^{2}\theta +\frac{{sin}^{4}\theta }{{Y}^{2}}\right]}^{-1/2},$$
  where ${W}_{sf}$ is shear strength of composite; ${W}_{sf}={P}_{{45}^{^\circ }}/2 b t$ and ${P}_{{45}^{^\circ }}$ denotes ultimate bearing capacity of the test coupon with 45° printing direction. Moreover, b and t indicate width and thickness of the specimen, respectively.
- Second model: Based on the Eqs. (10) and E(11), the UTS of loading direction can be determined as follows:
  13 $${T}_{\theta }={\left[\frac{{cos}^{4}\theta }{{X}^{2}}+\left(\frac{1}{{W}_{HT}}-\frac{1}{{X}^{2}}\right){sin}^{2}\theta {cos}^{2}\theta +\frac{{sin}^{4}\theta }{{Y}^{2}}\right]}^{-1/2}.$$

The last two equations, along with the UTS (X and Y) can be utilized to calculate the strength with respect to the printing angle.

## Experimental procedure

### Specimen preparation

In this study, PLA material was used for fabrication of the test coupons based on the FDM process. The specimens are designed according to ASTM D638^46^. Considering the aim of this research, two printing parameters were changed during printing of the specimens: (a) raster orientation, and (b) printing speed. These parameters are defined as follows:

- Raster orientation: the angle $(\alpha )$ indicates the printing direction relative to the loading direction.
- Printing speed: the distance traveled by the extruder along the bed per unit time while extruding. After design of the dog-bone shaped specimens, 3D models were transferred into a slicer software and the output was saved as ‘.stl’ format. Later, the models were printed utilizing FDM 3D printer. The printing parameters are presented in Table 2.

**Table 2:** Printing parameters and properties of fabricated specimens.

Parameters	Values
Nozzle temperature $(^\circ \mathrm{C})$	215
Bed temperature $(^\circ \mathrm{C})$	55
Printing speed (mm/s)	20, 80
Infill percentage (%)	100
Density (g/cm^3^)	1.24
Layer thickness (mm)	0.4
Number of contours	2
Nozzle diameter (mm)	0.8
Extrusion width (mm)	1
Raster angle $(^\circ )$	0$^\circ$, 30$^\circ$, 45$^\circ$, 60$^\circ$, 90$^\circ$

The specimens with unidirectional layup were printed in 8 layers with the total length of 160 mm and final thickness of 3.2 mm. The specimens were designed and fabricated with two contours (outer layer that is surrounding the internal structure).

In the present study, the dog-bone shaped specimens were printed under two different printing speeds: 20 mm/s and 80 mm/s. For each raster orientation and printing speed, six test coupons were fabricated. Later, the specimens were subjected to the tensile loads which is described in the following subsection.

### Mechanical tests

A series of tensile tests was performed by using a hydraulic mechanical testing machine under temperature and relative humidity of 23 ± 3 °C and 50 ± 5%, respectively. The machine The dog-bone shaped specimens with gauge length of 150 mm experienced quasi-static uniaxial tensile loads and their force–displacement curves were recorded at a constant crosshead speed of 5 mm/min. In the experimental practices, each examined specimen was fixed on the machine by an appropriate tensile fixture. It is noteworthy that, failure of the specimens was occurred at the gauge length of the test coupons. It means the documented breaking strength is accurately represent the actual breaking strength of the examined specimens. The 3D-printed specimens with different raster orientations underwent a series of tensile tests and the average of failure load and relevant displacements are presented in Table 3.

**Table 3:** The experimental failure loads and displacements of the examined 3D-printed specimens.

Material	Raster direction (°)	Printing speed (mm/s)	Failure load (N)	Displacement (mm)
PLA	0	20	5285.9	6.9
		80	5149.3	6.1
	30	20	4749.1	6.3
		80	4523.6	5.7
	45	20	4138.2	5.8
		80	3849.3	5.1
	60	20	3451.7	5.2
		80	3226.4	4.9
	90	20	2793.5	5.1
		80	2638.2	4.8

The obtained values presented in Table 3 showed that the failure load decreases while the raster angle is increased. Moreover, there is a gradual decrease in breaking force while printing speed is increased.

## Conclusion

Experimental results demonstrated that raster direction has a significant influence on the strength of the specimens. In fact, the highest elastic modulus belongs to 0° specimens. This value was gradually decreased with increase in the raster direction. Moreover, lowest ultimate tensile strength was reduced 53.76% in the specimen with the raster direction of 90° printed at 80 mm/s. In this study, we have documented that strength was decreased according to increase of printing speed. In detail, higher speed can reduce the extrusion volume which decreased printing stability. Although printing speed has minor effect on strength of specimens (compared to raster direction), it has a crucial role on cost of production. However, combination of raster direction and printing speed showed a crucial effect on the strength and mechanical behavior of 3D-printed test coupons. Based on the conducted fractographic analysis we have documented that fractures are oriented with raster direction. Since in this study a biopolymer PLA was investigated, the obtained results can be used for analysis on biocomposite which used PLA with same grade. The obtained data can be used for new designs, reinforcement configurations, and next computational models.

## Data Availability

The datasets generated during and/or analysed during the current study are available from the corresponding author on reasonable request.
